# Electrolyte tuning in dye-sensitized solar cells with *N*-heterocyclic carbene (NHC) iron(II) sensitizers

**DOI:** 10.3762/bjnano.9.285

**Published:** 2018-12-21

**Authors:** Mariia Karpacheva, Catherine E Housecroft, Edwin C Constable

**Affiliations:** 1 Department of Chemistry, University Basel, BPR 1096, Mattenstrasse 24a, CH-4058 Basel, Switzerland

**Keywords:** dye-sensitized solar cell, electrolyte, nanoparticles, *N*-heterocyclic carbene iron(II) complex, solar energy conversion, sustainable energy

## Abstract

We demonstrate that the performances of dye-sensitized solar cells (DSCs) sensitized with a previously reported *N*-heterocyclic carbene iron(II) dye in the presence of chenodeoxycholic acid co-adsorbant, can be considerably improved by altering the composition of the electrolyte while retaining an I^−^/I_3_^−^ redox shuttle. Critical factors are the solvent, presence of ionic liquid, and the use of the additives 1-methylbenzimidazole (MBI) and 4-*tert*-butylpyridine (TBP). For the electrolyte solvent, 3-methoxypropionitrile (MPN) is preferable to acetonitrile, leading to a higher short-circuit current density (*J*_SC_) with little change in the open-circuit voltage (*V*_OC_). For electrolytes containing MPN, an ionic liquid and MBI (0.5 M), DSC performance depended on the ionic liquid with 1-ethyl-3-methylimidazolium hexafluoridophosphate (EMIMPF) > 1,2-dimethyl-3-propylimidazolium iodide (DMPII) > 1-butyl-3-methylimidazolium iodide (BMII) ≈ 1-butyl-3-methylimidazolium hexafluoridophosphate (BMIMPF). Omitting the MBI leads to a significant improvement in *J*_SC_ when the ionic liquid is DMPII, BMII or BMIMPF, but with EMIMPF the removal of the MBI additive results in a dramatic decrease in *V*_OC_ (542 to 42 mV). For electrolytes containing MPN and DMPII, the effects of altering the MBI concentration have also been investigated. Although the addition of TBP improves *V*_OC_, it causes significant decreases in *J*_SC_. The best performing DSCs with the NHC-iron(II) dye employ an I^−^/I_3_^−^-based electrolyte with MPN as solvent, DMPII ionic liquid (0.6 M) with no or 0.01 M MBI; values of *J*_SC_ = 2.31 to 2.78 mA cm^−2^, *V*_OC_ = 292 to 374 mV have been achieved giving η in the range of 0.47 to 0.57% which represents 7.8 to 9.3% relative to an N719 reference DSC set at 100%. Electrochemical impedance spectroscopy has been used to understand the role of the MBI additive in the electrolytes.

## Introduction

**Scheme 1:**
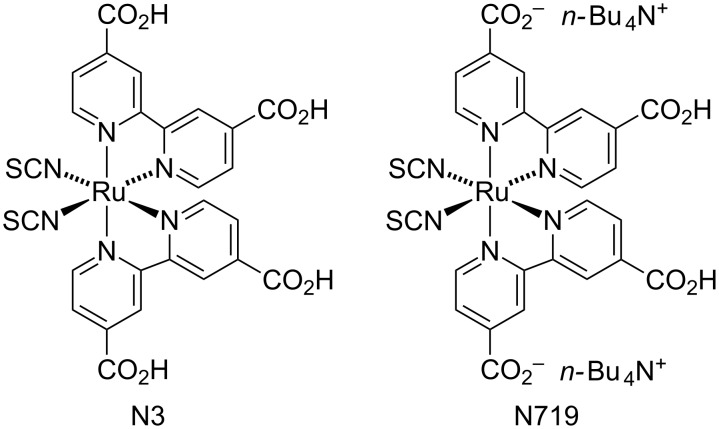
Structures of the ruthenium dyes N3 and N719.

The field of dye sensitized solar cells (DSCs) has developed significantly [1–3] since the pioneering publication of O’Regan and Grätzel [4]. Photoconversion efficiencies (η) of ≈11–14% have been realized with ruthenium-based [2], zinc(II) porphyrin-based [5–9] or metal-free organic dyes [10–12]. In a recent review [2], Nazeeruddin points to the fact that only incremental enhancements of the photoconversion efficiencies of ruthenium dyes have occurred during the last two decades, with N3 (first reported in 1993 [13]) and N719 [14] (Scheme 1) remaining the state-of-the-art dyes. The low natural abundance of ruthenium (≈0.001 ppm in the Earth's crust [15]) and its consequential high cost motivates thorough investigations of the use of dyes based on Earth-abundant metals. Among the first row *d*-block metals, iron, cobalt, nickel, copper and zinc have Earth-crustal abundances of ≈41,000, 20, 80, 50 and 75 ppm, respectively. We and others [16–19] have realized the potential of DSCs sensitized by bis(diimine)copper(I) complexes, and both we [20] and Dragonetti et al. [21] have demonstrated the viability of DSCs comprising both copper(I)-based dyes and copper(I)/copper(II) redox shuttles. Photoconversion efficiencies in the range of 3–5% have been achieved for DSCs containing copper(I) dyes [22–24]. However, based upon the 800-fold greater natural abundance of iron versus copper, the holy grail of sustainable DSCs is the use of iron-based sensitizers.

**Scheme 2:**
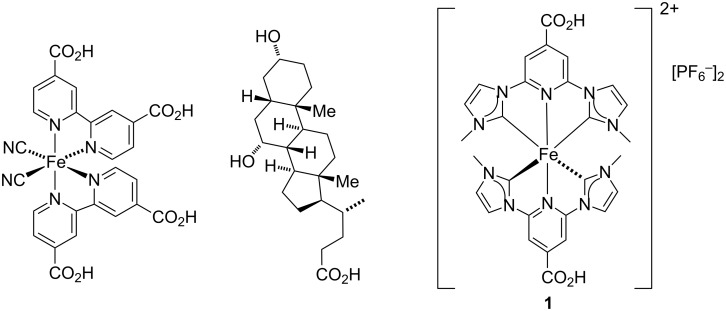
Structures of *cis*-[Fe{bpy-4,4'-(CO_2_H)_2_}_2_(CN)_2_], the co-adsorbant cheno, and the NHC iron(II) dye **1** used in this investigation.

Ferrere and Gregg [25] were the first to report of the use of a simple iron(II) sensitizer in a functional DSC; in the presence of the co-adsorbant chenodeoxycholic acid (cheno, Scheme 2), a DSC containing *cis*-[Fe{bpy-4,4'-(CO_2_H)_2_}_2_(CN)_2_] (Scheme 2) achieved a short-circuit current density (*J*_SC_) of 0.29 mA cm^−2^ and an open-circuit voltage (*V*_OC_) of 360 mV. However, little progress was made in this area [26] until the interest shifted to the use of iron(II) complexes incorporating *N*-heterocyclic carbene (NHC) ligands. Replacing polypyridyl ligands by NHCs leads to a remarkable increase in the lifetime of the ^3^MLCT excited state (1000× longer), considerably enhancing electron injection [27–33]. The homoleptic complex **1** (Scheme 2) is currently the best-performing iron(II) NHC sensitizer among a series of related complexes screened in n-type DSCs [32]. In MeCN solution, **1** exhibits an MLCT band at λ_max_ = 520 nm with an extinction coefficient, ε_max_, of 16,200 dm^3^ mol^−1^ cm^−1^ [33]. Herein lies an advantage of NHC iron(II) complexes over bis(diimine)copper(I) dyes, since the latter absorb in the visible with values of ε_max_ of ≈5,000 dm^3^ mol^−1^ cm^−1^ [16]. Gros and co-workers have demonstrated that a DSC sensitized with **1** in the presence of the co-adsorbant cheno (Scheme 2) and with an I^−^/I_3_^−^ redox couple in MeCN achieves values of *J*_SC_ = 0.41 mA cm^−2^, *V*_OC_ = 457 mV, fill factor (ff) = 68% and η = 0.13%. These parameters compare with *J*_SC_ = 13.25 mA cm^−2^, *V*_OC_ = 687 mV, ff = 67% and η = 6.1% for a reference DSC containing N719 [34]. Previous DSC investigations have focused on structurally modifying the NHC iron(II) complex while maintaining a common co-adsorbant and electrolyte [32,34]. It is well established for ruthenium(II), copper(I) and organic dyes that modification of the electrolyte can have a profound effect on DSC performance [17,35–39]. We now demonstrate the effects on the performance of DSCs sensitized by the NHC iron(II) dye **1** of varying the solvent or additives in an I^−^/I_3_^−^-based electrolyte. We present data for fully masked DSCs to avoid overestimation of their performance [40].

## Results and Discussion

### Effects of solvent and ionic liquid

**Scheme 3:**
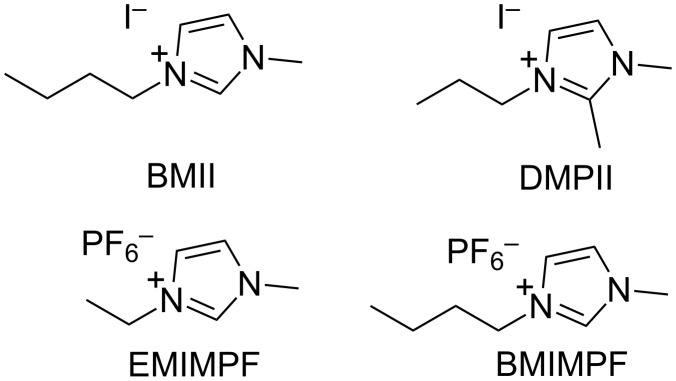
Structures of ionic liquids.

The working electrodes for the DSCs were prepared using commercial FTO/TiO_2_ electrodes immersed in a MeCN solution containing a mixture of sensitizer **1** and the co-adsorbant cheno (see Experimental section). Duplicate cells were made for all measurements to ensure reproducibility of the data. An I^−^/I_3_^−^ redox couple was used in all DSCs, and the electrolyte compositions for the first two series of investigations are given in Table 1. In typical I^−^/I_3_^−^-based electrolytes, LiI and I_2_ are present in a solvent such as MeCN, 3-methoxypropionitrile (MPN) or valeronitrile, with added ionic liquid, often 1-butyl-3-methylimidazolium iodide (BMII, Scheme 3). Additives are also present to tune the TiO_2_ conduction band energy and reduce detrimental recombination of electrons and oxidized dye [41–44].

**Figure 1:**
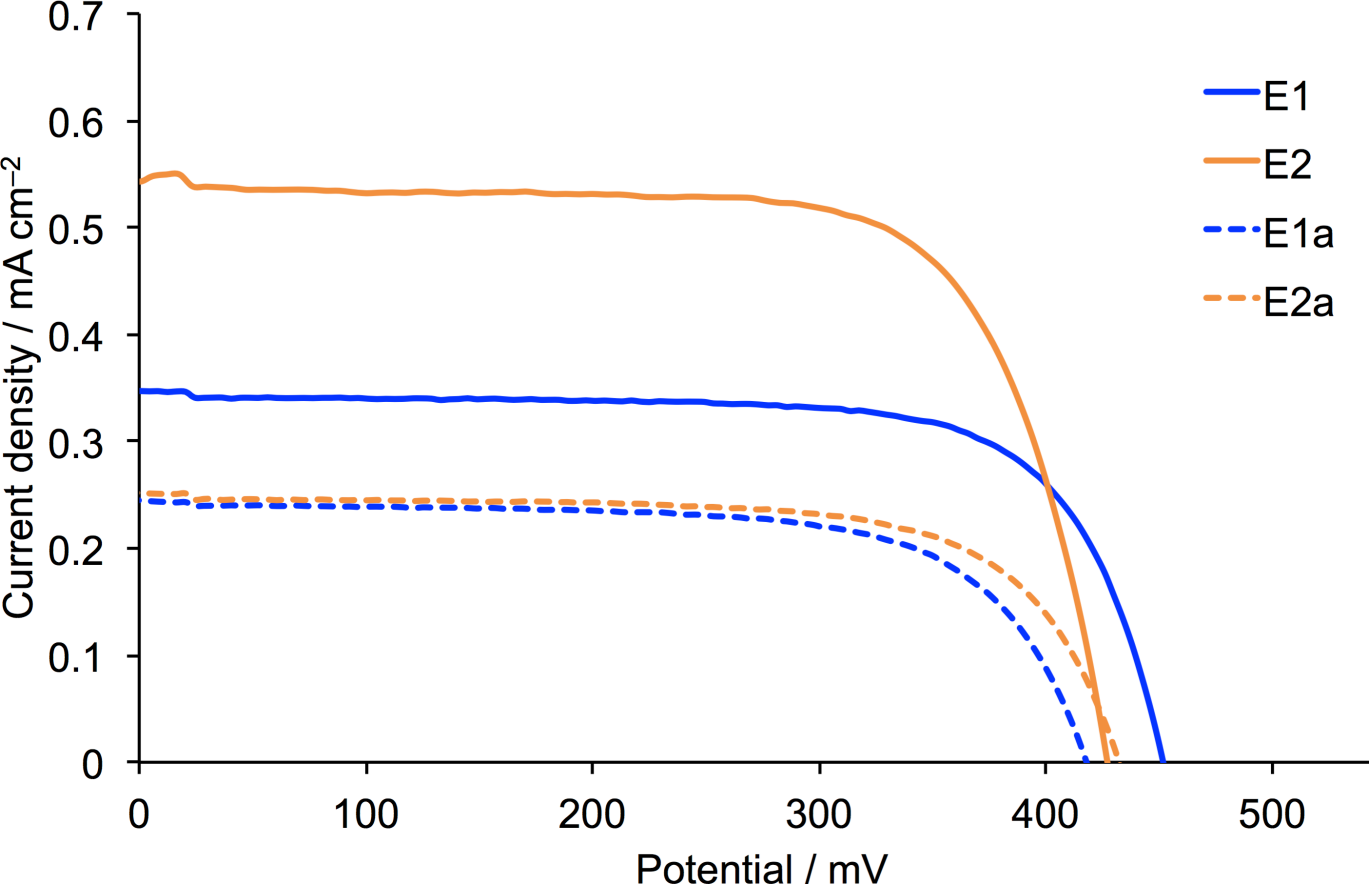
*J–V* curves for DSCs containing dye **1** and electrolytes with MPN (E1, E2) and MeCN (E1a, E2a) as solvents.

We initially focused on the effect of changing the electrolyte solvent from MeCN (present in the commercial Solaronix electrolyte AN-50 used by Gros and co-workers [34]) to the more viscous and higher boiling MPN. The choice of solvent is crucial for optimization of DSC performance and long-term stability [45–46]. Our starting point was the standard electrolyte (E1, Table 1) that we employ for DSCs with bis(diimine)copper(I) sensitizers. This electrolyte contains the additive *N*-methylbenzimidazole (MBI) which is known to enhance values of *V*_OC_ [47]. Electrolytes E1 and E1a differ only in a change in solvent from MPN to MeCN (Table 1), while on going from E1 to E2 (or E1a to E2a), the ionic liquid is changed from 1-butyl-3-methylimidazolium iodide (BMII) to 1,2-dimethyl-3-propylimidazolium iodide (DMPII, Scheme 3). The latter is also present in the commercial electrolyte AN-50. The performance parameters and *J*–*V* curves for DSCs sensitized with the NHC iron(II) dye **1** and containing electrolytes E1, E1a, E2 and E2a are shown in Table 2 and Figure 1. The data in Table 2 are referenced with respect to a DSC sensitized with N719 (Scheme 1) and in the right-hand column of Table 2, relative efficiencies are given with η(N719) set to 100%. Data for multiple cells confirm the reproducibility of the DSC performances (Table S1, Supporting Information File 1).

**Table 1:** Compositions of electrolytes E1, E1a, E2, E2a, E3 and E4.

Electrolyte	[LiI]	[I_2_]	Ionic liquid^a^	Additive	Solvent

E1	0.1 M	0.05 M	BMII 0.6 M	MBI 0.5 M	MPN
E2	0.1 M	0.05 M	DMPII 0.6 M	MBI 0.5 M	MPN
E1a	0.1 M	0.05 M	BMII 0.6 M	MBI 0.5 M	MeCN
E2a	0.1 M	0.05 M	DMPII 0.6 M	MBI 0.5 M	MeCN
E3	0.1 M	0.05 M	BMIMPF 0.6 M	MBI 0.5 M	MPN
E4	0.1 M	0.05 M	EMIMPF 0.6 M	MBI 0.5 M	MPN

^a^Abbreviations are defined in the text.

**Table 2:** Parameters for masked DSCs using electrolytes with different ionic liquids with MPN and MeCN as solvent. Measurements^a^ were made on the day of sealing the cells. See Supporting Information File 1, Table S1 for data for multiple DCSs. Data are referenced with respect to a DSC with N719.

Electrolyte	*J*_SC_ [mA cm^−2^]	*V*_OC_ [mV]	ff [%]	η [%]	Rel. η [%]

E1	0.34	451	73	0.11	1.8
E2	0.54	427	71	0.17	2.8
E1a	0.24	418	68	0.07	1.2
E2a	0.25	432	69	0.07	1.2
E3	0.29	480	73	0.10	1.6
E4	0.82	542	69	0.31	5.1
N719	13.87	705	62	6.02	100

^a^*J*_SC_ = short-circuit current density; *V*_OC_ = open-circuit voltage; ff = fill factor; η = photoconversion efficiency; Rel. η = η relative to N719 set to 100%.

**Figure 2:**
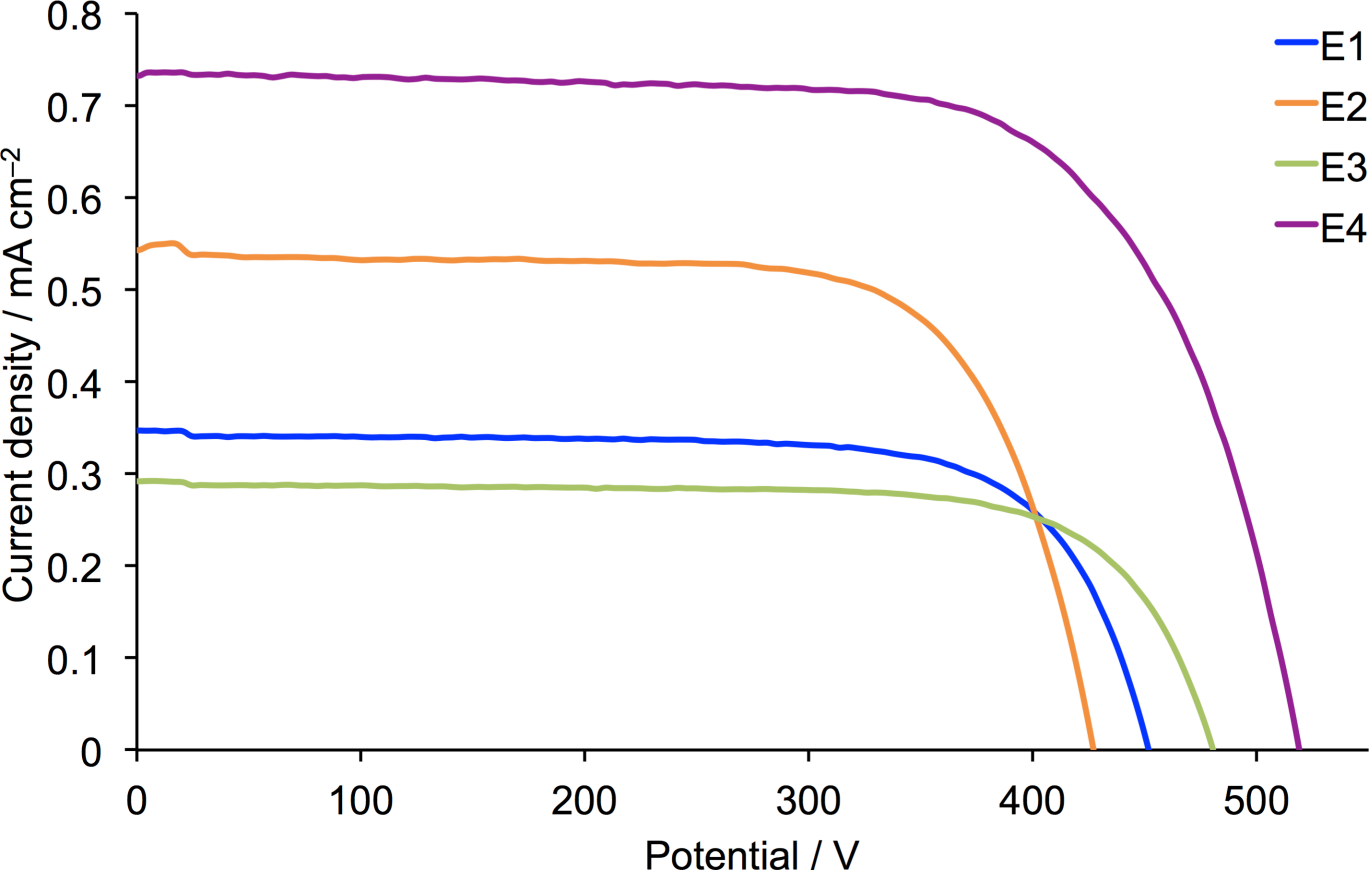
*J–V* curves for DSCs containing dye **1** electrolytes E1–E4 with different ionic liquids. The solvent is MPN.

We first note that all DSCs exhibit good fill factors, indicating satisfactorily operating devices. The performances of the masked DSCs with electrolytes E1a and E2a (MeCN as solvent) are slightly lower than those reported by Gros [34], consistent with the masking [40] of the DSCs in the present study. Significantly, a change from MeCN to MPN enhances *J*_SC_ with little change in *V*_OC_ or the fill factor, and results in a gain in global efficiency. A further gain is observed upon changing the ionic liquid with values of *J*_SC_ in the order E4 > E2 > E1 ≈ E3 (Figure 2, Table 2 and Supporting Information File 1, Table S1). The use of EMIMPF in electrolyte E4 enhances both *J*_SC_ and *V*_OC_. The improved performance observed with electrolyte E4 was verified for a set of four DSCs, with values of *J*_SC_ and *V*_OC_ in the ranges of 0.70–0.90 mA cm^−2^ and 519–542 mV, respectively (Table S1, Supporting Information File 1).

### Effects of additives

Each of electrolytes E1–E4 contains MBI (0.5 M), and we next investigated the electrolytes without this additive. Electrolytes E1b–E4b are compositionally analogous to E1–E4 but without MBI (Table 3). Comparison of the parameters in Table 2 and Table 4 demonstrates the effects of eliminating MBI. On-going from E1 to E1b, E2 to E2b, or E3 to E3b, an increase in *J*_SC_ coupled with a decrease in *V*_OC_ is observed resulting in an overall enhancement in *η*. For E2b, the efficiency relative to N719 of 8.5% (Table 4) is remarkable for an iron(II)-based sensitizer, and arises from a significantly enhanced value of *J*_SC_. The result was verified using four DSCs (Table S3, Supporting Information File 1) for which values of *J*_SC_ in the range 2.31–2.58 mA cm^−2^ and relative efficiencies of 7.8–9.5% were observed. However, the improvement in *J*_SC_ is at the expense of *V*_OC_ (292–374 mV for electrolyte E2b versus 426–427 mV for E2, Table S1 and Table S3, Supporting Information File 1).

**Table 3:** Electrolyte compositions, showing the changes in additives in electrolytes E1–E4.

Electrolyte	[LiI]	[I_2_]	Ionic liquid^a^	Additive	Solvent

E1b	0.1 M	0.05 M	BMII 0.6 M	–	MPN
E2b	0.1 M	0.05 M	DMPII 0.6 M	–	MPN
E3b	0.1 M	0.05 M	BMIMPF 0.6 M	–	MPN
E4b	0.1 M	0.05 M	EMIMPF 0.6 M	–	MPN
E2c	0.1 M	0.05 M	DMPII 0.6 M	MBI 0.1 M	MPN
E2d	0.1 M	0.05 M	DMPII 0.6 M	MBI 0.05 M	MPN
E2e	0.1 M	0.05 M	DMPII 0.6 M	MBI 0.01 M	MPN
E2f	0.1 M	0.05 M	DMPII 0.6 M	TBP 0.1 M	MPN
E2g	0.1 M	0.05 M	DMPII 0.6 M	TBP 0.05 M	MPN
E2h	0.1 M	0.05 M	DMPII 0.6 M	TBP 0.5 M	MPN

^a^Abbreviations are defined in the text.

**Table 4:** Parameters for masked DSCs using different additives and additive concentrations (see Table 3) in the electrolytes. Measurements were made on the day of sealing the cell. See Table S2 and Table S3 (Supporting Information File 1) for multiple cell data.

Electrolyte	*J*_SC_ [mA cm^−2^]	*V*_OC_ [mV]	ff [%]	η [%]	Rel. η [%]

E1b	1.94	304	64	0.38	6.3
E2b	2.31	339	65	0.51	8.5
E3b	2.13	308	56	0.37	6.1
E4b	0.74	43	26	0.01	0.2
E2c	0.69	395	65	0.18	2.9
E2d	1.31	395	60	0.31	5.1
E2e	2.61	315	62	0.51	8.5
E2f	0.69	387	67	0.18	2.9
E2g	1.16	380	65	0.29	4.8
E2h	0.76	541	62	0.26	4.3
N719	13.87	705	62	6.02	100

**Figure 3:**
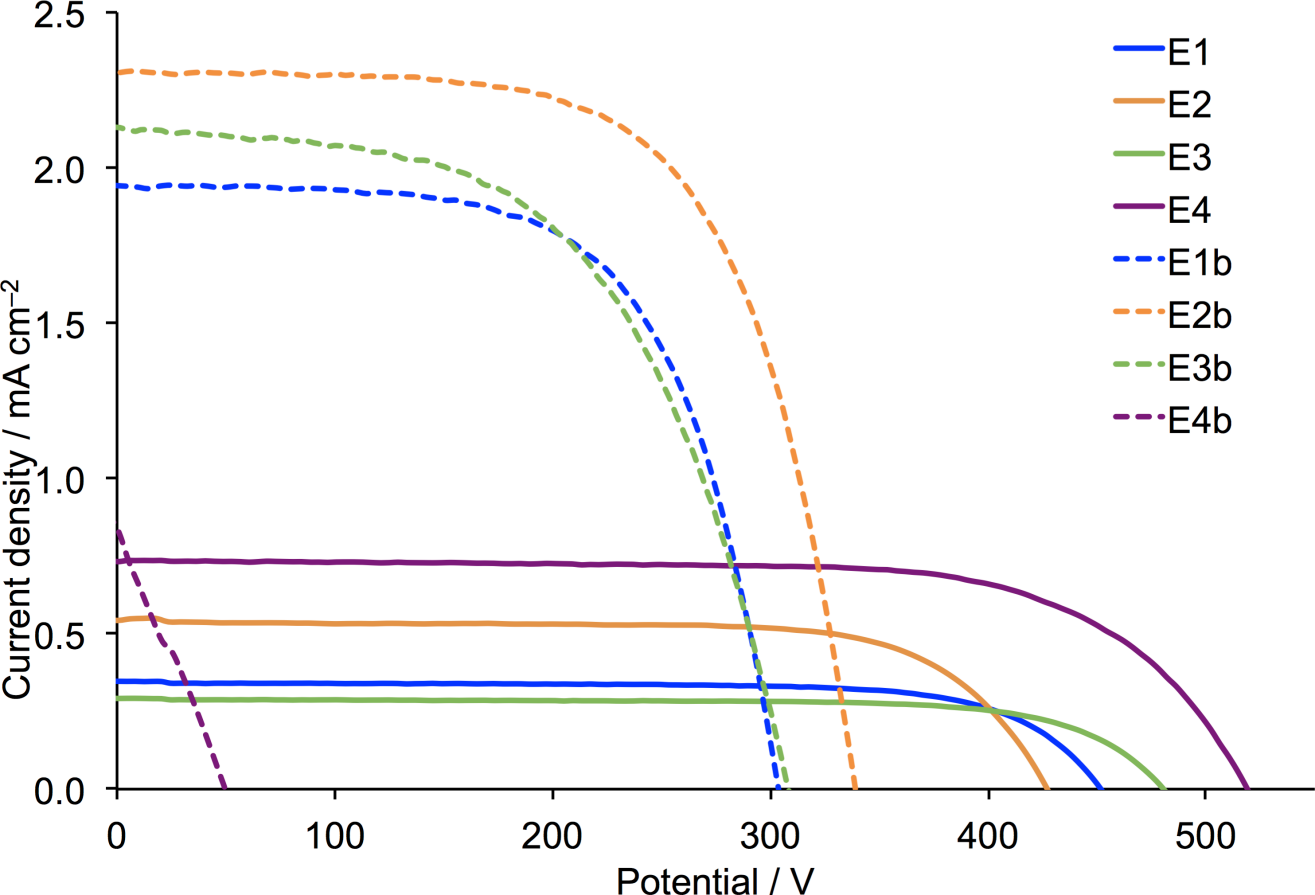
*J–V* curves to illustrate the effects of removing the MBI additive from electrolytes E1–E4.

The absence of the additive MBI from electrolyte E4b causes bleaching of the electrode when the dye-functionalized electrode surface comes in contact with the electrolyte; the original red colour of the dye-functionalized electrode becomes almost white. This has a dramatic effect on the value of *V*_OC_, with a fall from 542 to 42 mV (Table 2 and Table 4). The decrease was verified using duplicate DSCs (Supporting Information File 1, Table S1 and Table S2). While we have no clear explanation of this phenomenon, we note with interest that Dyson and co-workers have observed extensive interactions between imidazolium cations and polyiodide anions in ionic liquids doped with I_2_ [48] and this may be relevant to the use of ionic liquids in DSCs. The *J–V* curves in Figure 3 illustrate the effects on the performance of sensitizer **1** by using electrolytes with (E1–E4) and without (E1b–E4b) the additive MBI.

**Figure 4:**
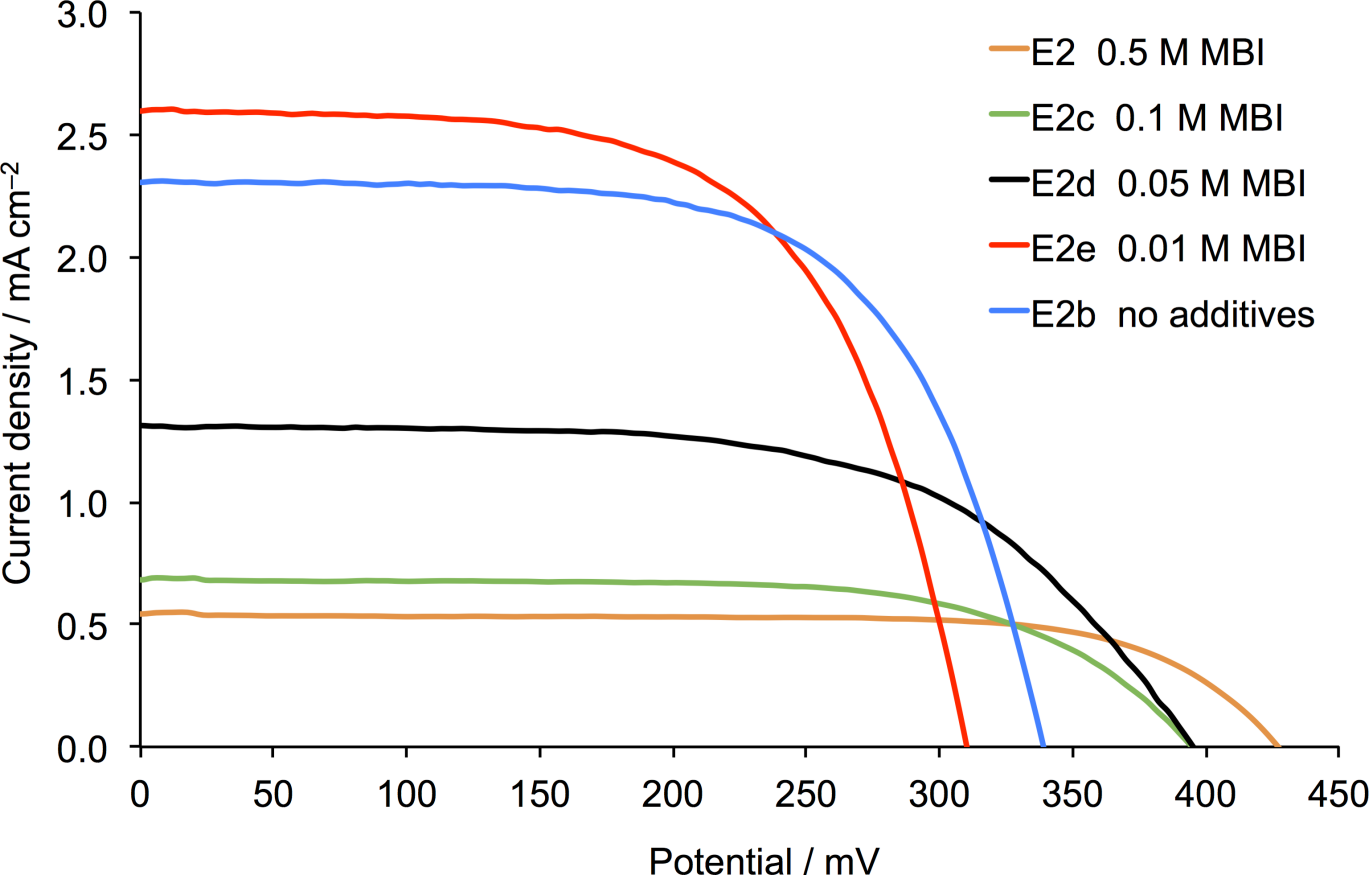
*J–V* curves for DSCs with dye **1** and electrolytes E2 and E2b–E2e. The curves were recorded on the day of sealing the DSCs.

**Figure 5:**
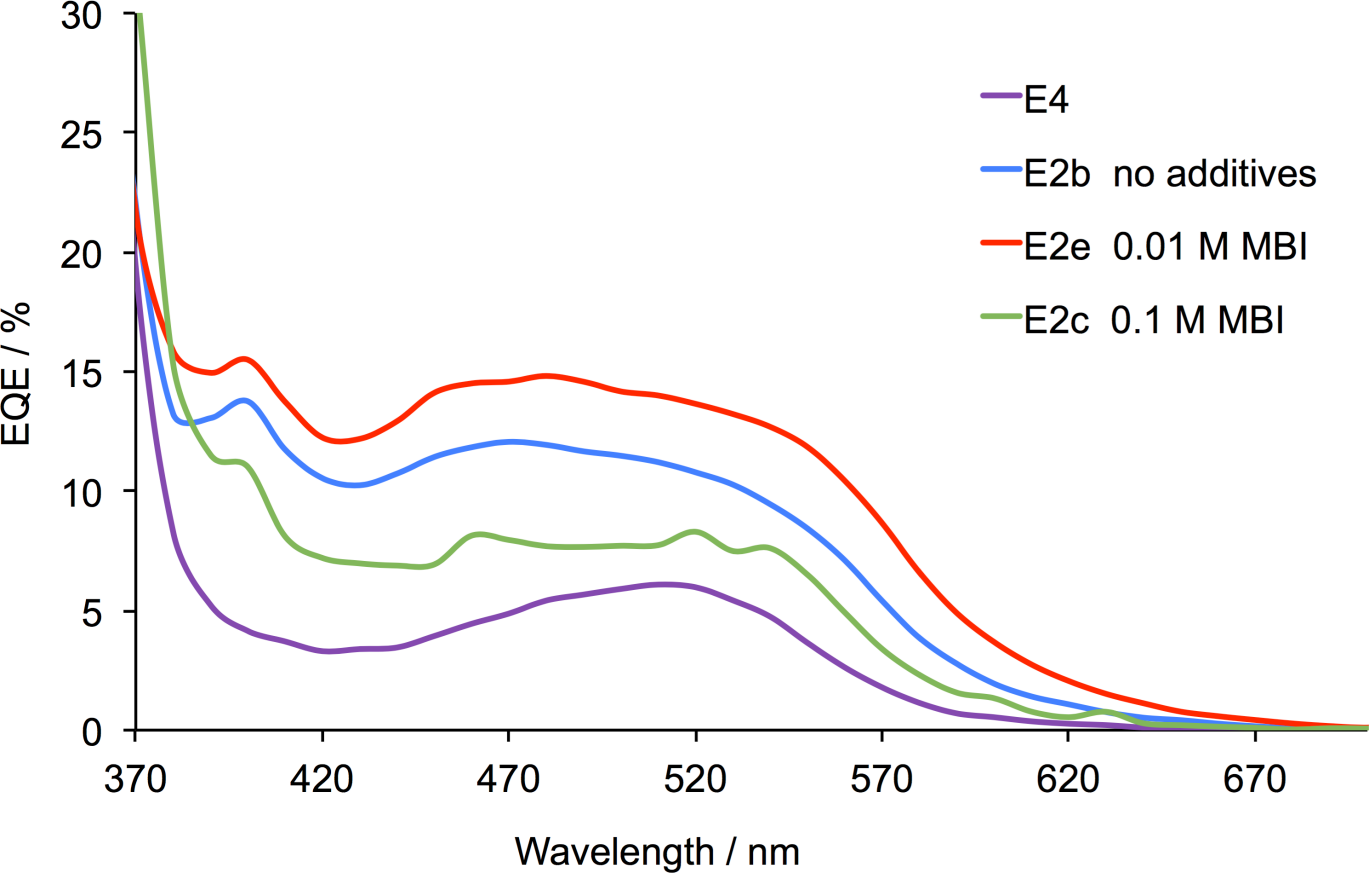
EQE spectra for the DSCs with dye **1** and electrolytes E4 (with EMIMPF as ionic liquid) and E2b, E2c, E2e (each with DMPII as ionic liquid) recorded on the day of sealing the DSCs. See also Figure S1, Supporting Information File 1.

The promising performances of DSCs with dye **1** combined with electrolyte E2b encouraged us to tune the components further. In Table 3, electrolytes E2c–E2h and E4f–E4h are based on E2 and E4 with different concentrations of MBI or TBP. We first consider MBI. The effects of altering the electrolyte composition with respect to MBI are seen in the *J–V* curves in Figure 4 and in the DSC parameters in Table 4 and Supporting Information File 1, Tables S1–S3. The general trend in Figure 4 is for an increase in *J*_SC_ as the concentration of MBI decreases: E2 with 0.5 M MBI gives the lowest *J*_SC_ (0.54 mA cm^−2^) but this is enhanced to >2.3 mA cm^−2^ by reducing the concentration of MBI to 0.01 M or removing the additive altogether. The data for multiple DSCs in Table S3 confirm the observation with values of *J*_SC_ in the range 2.31–2.58 mA cm^−2^ for electrolyte E2b (no MBI) and 2.61–2.78 mA cm^−2^ for electrolyte E2e (0.01 M MBI). DSCs with these two electrolytes exhibit values of *V*_OC_ in the range 292–374 mV (Supporting Information File 1, Table S3) and overall photoconversion efficiencies of 0.47–0.57% (or 7.8–9.5% with respect to N719 set to 100%). External quantum efficiency (EQE) curves are shown in Figure 5 and Figure S1 (Supporting Information File 1). The EQE spectra are broad with λ_max_ in the range of 480–520 nm, consistent with the incident photon to current efficiency (IPCE) λ_max_ of 500 nm reported by Gros [34]. The use of DMPII rather than EMIMPF in the electrolyte enhances the value of EQE_max_ from ≈6 to 15%. The highest EQE_max_ values of ≈12% for electrolyte E2b and ≈15% for E2e are consistent with the trends in *J*_SC_ discussed above. These values compare with EQE_max_ ≈ 2.3% for a DSC sensitized with **1** and using the commercial AN-50 electrolyte [34].

**Figure 6:**
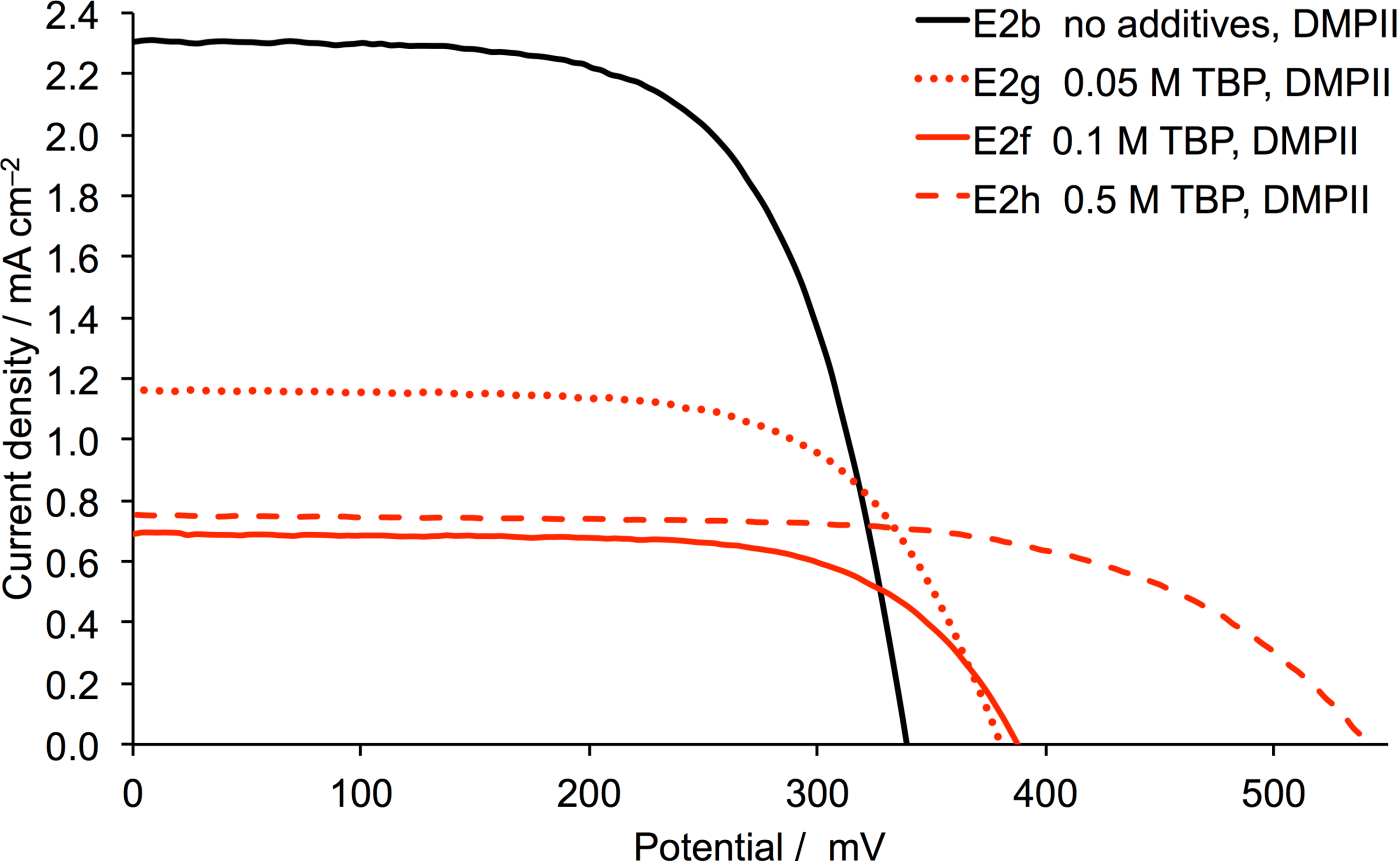
*J–V* curves for DSCs with dye **1** and electrolytes with TBP additive.

We now turn to the effects of using TBP as an additive, while retaining DMPII as the ionic liquid in the electrolyte. For ruthenium dyes such as N719 combined with an I^−^/I_3_^−^ redox couple, it is well established that TBP leads to improved open-circuit voltage [44]. On the other hand, we have previously demonstrated that for a representative heteroleptic bis(diimine)copper(I) dye, the addition of TBP to a standard I^−^/I_3_^−^-based electrolyte in MPN is detrimental to DSC performance [38]. In the current investigation, electrolytes E2f, E2g and E2h were prepared with different concentrations of TBP as additive (Table 3). The *J–V* curves shown in Figure 6 and the DSC parameters in Table 4 and Table S2 (Supporting Information File 1) demonstrate a significant decrease in *J*_SC_ even when TBP is present in 0.05 M concentration. A further increase in the concentration lowers *J*_SC_ further while the value of *V*_OC_ increases from 339 mV (no TBP) to 541 mV (0.5 M TBP). However, this gain is not sufficient to enhance the global efficiency which drops from 0.51% to 0.26% (Table 4). The trends are reproduced for duplicate DSCs (Table S2, Supporting Information File 1).

### Electrochemical impedance spectroscopy (EIS)

Electrochemical impedance spectroscopy (EIS) is an important technique for the investigation of interfaces in DSCs [49–50]. Fitting of the Nyquist and Bode plots, which are used to describe the EIS results, leads to parameters including the recombination resistance (*R*_rec_), electron/hole transport resistance (*R*_tr_), charge-transfer resistance at the counter electrode (*R*_Pt_) and the active layer surface chemical capacitance (*C*_µ_). All experiments in the following discussion were performed at *V*_OC_ conditions. The equivalent circuit model used in this study consisted of five elements (Figure S2, Supporting Information File 1). A series resistance (*R*_s_), a resistance (*R*_Pt_) and a constant phase element (CPE1) to model a platinum counter electrode, an extended distributed element (DX1) which represented the TiO_2_/electrolyte interface as a transmission line model, and a Warburg element (W_s_) associated with diffusion of the electrolyte. The constant phase element was employed in this study because of the surface roughness [51–52].

**Figure 7:**
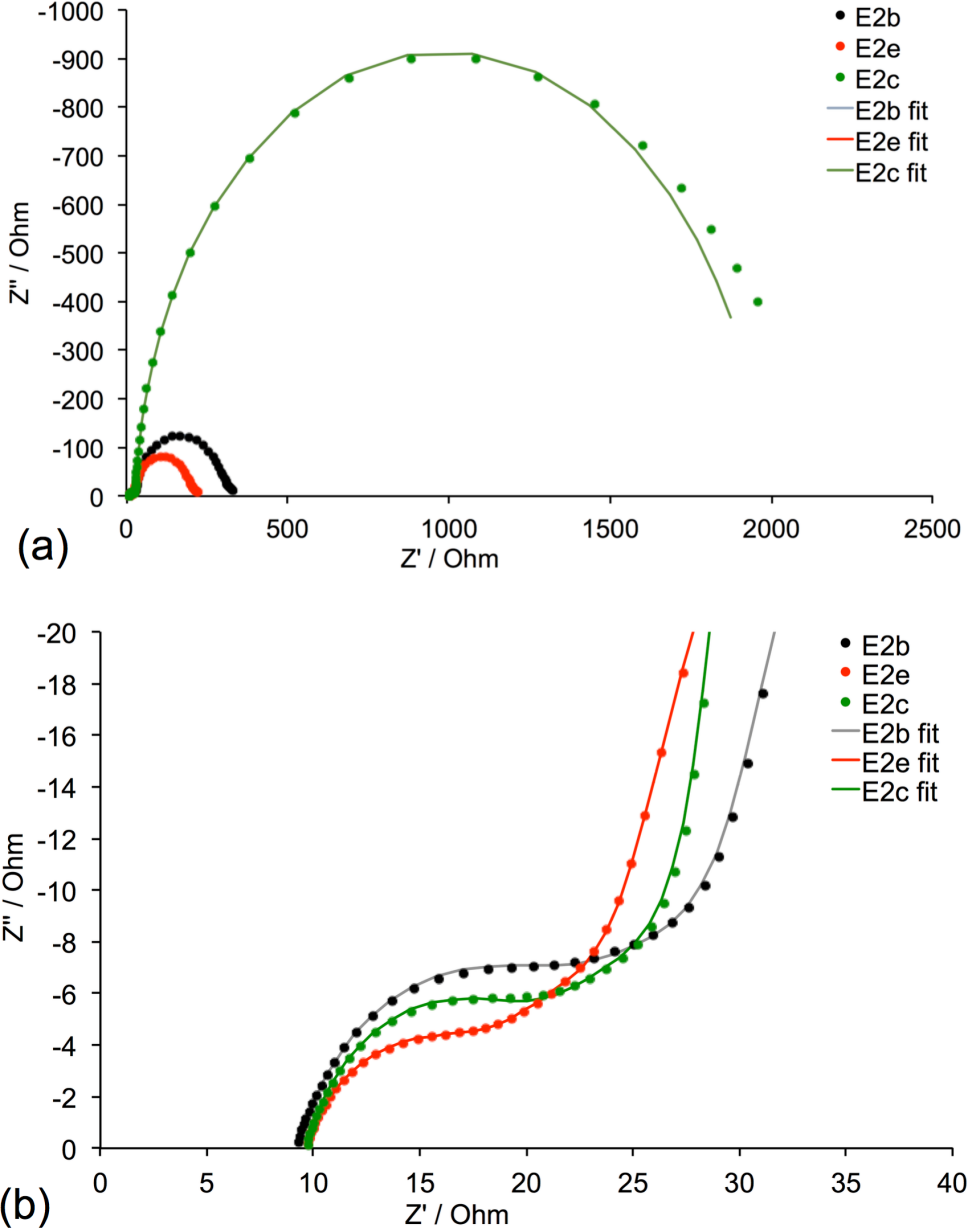
(a) Nyquist plots of DSCs with E2b, E2e and E2c electrolytes. (b) Expansion of the high frequency region. Experimental data are represented by dots and solid lines represent fitted results.

We chose to focus on understanding the observations involving the MBI additive, and EIS studies were conducted for electrolytes E2b, E2c and E2e. Measurements and curve fitting were made for duplicate cells to confirm the trends discussed below; data for one cell for each electrolyte are presented. The key parameters of the EIS measurements are summarized in Table 5, and the Nyquist plots are shown in Figure 7. The series resistance (*R*_S_, measured from zero to the start of the first semi-circle in the plot and arising from the charge resistance at the FTO/TiO_2_ interface [53]) is constant (≈9 Ω) for the three DSCs (Figure 7b). The value of *R*_Pt_ is extracted from the first semi-circle in the Nyquist plot (Figure 7b) and Table 5 shows these values to be similar for all DSCs. In principle, two further semi-circles should be observed in the Nyquist plot, one corresponding to *R*_rec_ and one to the diffusion resistance of the charge carriers in the electrolyte (*R*_d_). However, as seen in Figure 7, these may overlap. The most striking feature of Figure 7 is the far larger second semi-circle for the DSC with electrolyte E2c, corresponding to a very significant increase in *R*_rec_ (Table 5) when 0.1 M MBI was used. EIS measurements were also made for a DSC with electrolyte E2 containing 0.5 M MBI but we were unable to fit the data because of the extreme dominance of the second semicircle associated with an extremely high recombination resistance.

**Table 5:** EIS data obtained from measurements at a light intensity of 22 mW cm^−2^ of n-type DSCs containing FTO/TiO_2_ working electrodes, dye **1** and either electrolytes E2b, E2e or E2c (see Table 3 for compositions). Experimental values of *J*_SC_, *V*_OC_ and η from Table 4 are included for convenience.

Electrolyte	*R*_rec_ [Ω]	*C*_μ_ [μF]	*R*_tr_ [Ω]	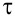 [ms]	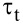 [ms]	*L*_d_ [μm]	*R*_s_ [Ω]	*R*_Pt_ [Ω]	*C*_Pt_ [μF]	*J*_SC_ [mA cm^−2^]	*V*_OC_ [mV]	η [%]

E2b (no MBI)	254.6	233.1	30.0	59.3	6.9	34.9	8.9	9.3	7.4	2.31	339	0.51
E2e (0.01 M MBI)	138.3	321.9	25.8	44.5	8.3	27.8	9.4	5.7	8.6	2.61	315	0.51
E2c (0.1 M MBI)	1830	306.7	25.3	561.3	7.8	102.1	9.5	7.7	8.1	0.69	395	0.18

The electrolytes E2b and E2e with no MBI and 0.01 M MBI, respectively, (Table 3) have the same overall photoconversion efficiencies (η = 0.51%, Table 4) and similar values of *J*_SC_ (2.31 and 2.61 mA cm^−2^). In contrast, a ten-fold increase in the concentration of MBI to 0.1 M leads to significantly lower *J*_SC_ (0.69 mA cm^−2^) and poor DSC efficiency (η = 0.18%). Compared to electrolyte E2b with no additive, the presence of 0.01 M MBI in electrolyte E2e leads to a lower *R*_rec_, a higher *C*_µ_ and a lower *R*_tr_. A combination of higher capacitance and lower transport resistance is consistent with a higher value of *J*_SC_ (Table 5). Combinations of lower *C*_μ_ and higher *R*_rec_ for E2b and higher *C*_μ_ and lower *R*_rec_ for E2e lead to similar DSC performances.

Greater recombination resistance and low *J*_SC_ values are observed when fewer electrons are injected to the semiconductor. Comparable *R*_tr_ values, but extremely high *R*_rec_ values for E2c lead to low *J*_SC_ and, as a consequence, to a low photoconversion efficiency. The value of the electron lifetime (τ) and the diffusion length (*L*_d_) are also important parameters for understanding the electron injection behaviour and these depend on the *R*_rec_ value [54]. A larger *L*_d_ and a longer lifetime as observed for electrolyte E2c result, respectively, in a higher electron density and lower charge loss in the semiconductor. For each electrolyte, the transport time (τ_t_) is lower than τ, resulting in efficient electron transport through TiO_2_. Trends in the electron lifetime, the diffusion length and the transport time are consistent with the *V*_OC_ values (Table 5) which increase in the order E2c > E2b > E2e.

### DSC stability

For electrolytes E2b and E2e, DSC performances were measured over a 40 day period and demonstrated the stability of the devices. Figure S3 (Supporting Information File 1) shows the trend in overall efficiencies which were the result of a general gain in *V*_OC_ (Figure S4, Supporting Information File 1) compensating for the general decrease in *J*_SC_ over time (Figure S5, Supporting Information File 1).

## Conclusion

**Figure 8:**
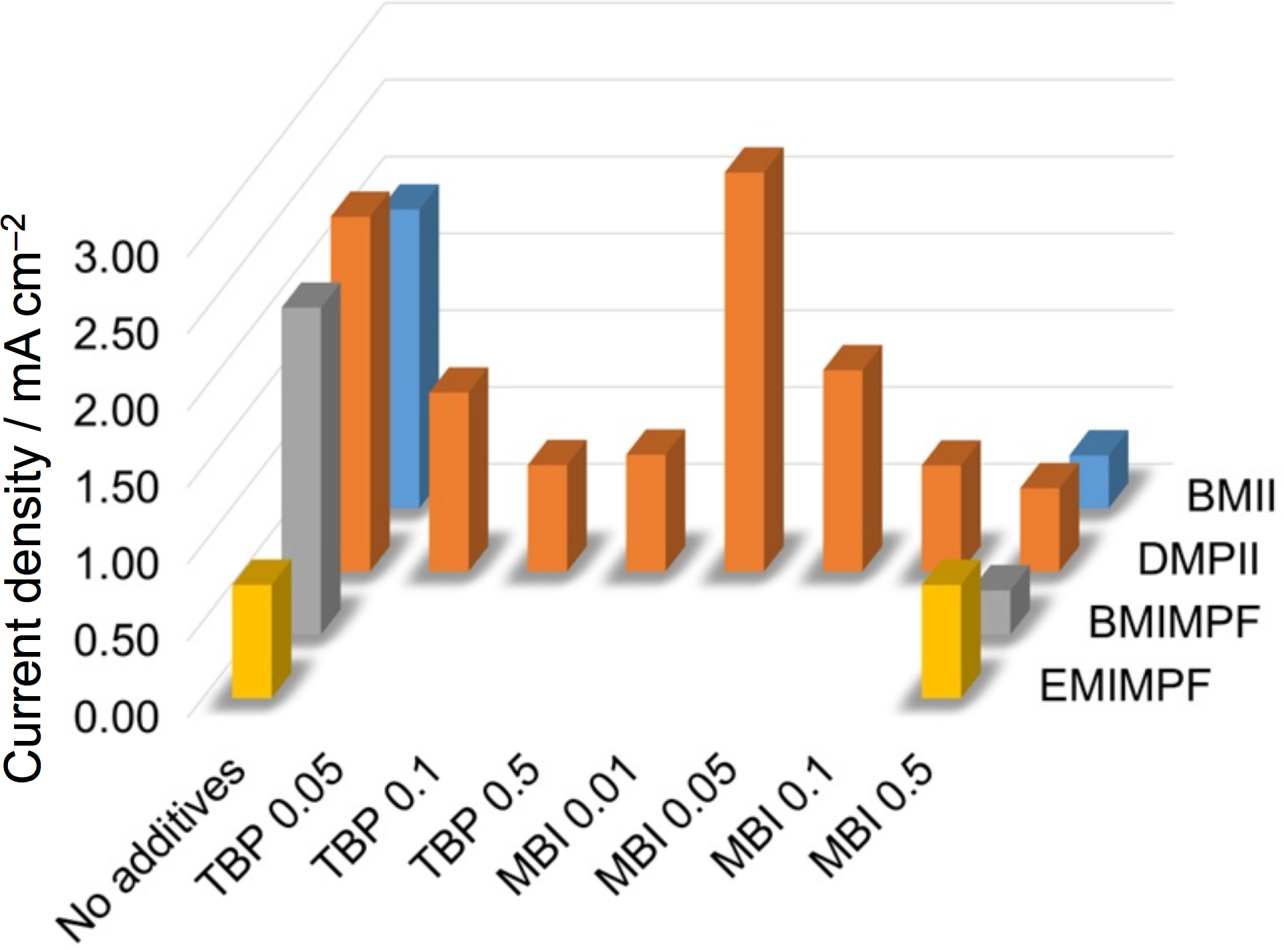
The dependence of short-circuit current density of the DSCs (on day of sealing the cells) on additives (concentrations in M). Electrolyte compositions are LiI (0.1 M), I_2_ (0.05 M), ionic liquid (0.6 M) in MPN with additives as specified on the abscissa.

We have shown that the performances of DSCs sensitized with the NHC iron(II) dye **1** originally reported by Gros and co-workers [34], can be significantly enhanced by tuning of the electrolyte composition, while retaining an I^−^/I_3_^−^ redox shuttle. The use of MPN in place of MeCN as solvent in the electrolyte leads to an improvement in *J*_SC_ with little change in *V*_OC_, resulting in enhanced photoconversion efficiency. The choice of the ionic liquid in the electrolyte is critical. Of the four ionic liquids investigated and with the additive MBI present at a concentration of 0.5 M, the order of DSC performance in terms of ionic liquid is EMIMPF > DMPII > BMII ≈ BMIMPF (right side of Figure 8). Removal of the MBI additive is highly detrimental to the DSC performance if the ionic liquid is EMIMPF with *V*_OC_ falling from 542 to 42 mV (Figure S6, Supporting Information File 1), but in contrast, leads to a significant improvement of *J*_SC_ values for DSCs in which the ionic liquid is DMPII, BMII or BMIMPF (Figure 8, left vs right sides). The addition of TBP is detrimental to *J*_SC_ (Figure 6 and Figure 8) while improving *V*_OC_ (Figure 6 and Figure S3, Supporting Information File 1). Further tuning of the electrolyte composition with MPN solvent and DMPII ionic liquid revealed that DSCs with 0.1 M MBI in the electrolyte performed slightly better than when MBI was absent. The best values of *J*_SC_ and *V*_OC_ were in the ranges of 2.31 to 2.78 mA cm^−2^, and 292 to 374 mV, respectively, leading to photoconversion efficiencies in the range of 0.47 to 0.57%. These represent 7.8 to 9.3% relative to an N719 reference DSC set at 100%. Our investigation has shown clearly that tuning of the components of electrolytes with an I^−^/I_3_^−^ redox shuttle is critical to progress in the field of NHC iron(II) sensitizers.

## Experimental

### General

Dye **1** was prepared as previously reported [31].

### Solar cell fabrication

Commercial titania electrodes (opaque, Solaronix) were used for the working electrodes. Each was rinsed with EtOH and dried on a heating plate at 500 °C for 30 min. The electrodes were cooled to 60 °C and immersed in a MeCN solution (0.5 mM) of the iron(II) dye **1** containing (0.1 mM) chenodeoxycholic acid overnight. Each reference working electrode was made by dipping a commercial titania electrode in an EtOH solution (0.3 mM) of dye N719 (Solaronix) overnight. After soaking in the dye baths, the electrodes were removed, washed with the same solvent as used in the dye bath (in case of MeCN the electrodes were washed a second time with acetone) and dried with a heat gun.

Commercial counter electrodes from Solaronix (Test Cell Platinum Electrodes Drilled) were rinsed with EtOH and dried on a heating plate at 500 °C for 30 min. The TiO_2_ electrodes and Pt counter-electrodes were combined using thermoplast hot-melt sealing foil (Solaronix, Test Cell Gaskets, made from Meltonix 1170-60 sealing film, 60 microns thick) by heating while pressing them together. The electrolyte was introduced into the DSC by vacuum backfilling through a hole drilled in the counter electrode and this was then sealed using hot-melt sealing foil and a cover glass.

The solar cell measurements used fully masked cells using a black coloured copper sheet with a single aperture placed over the screen printed dye-sensitized TiO_2_ square. The area of the aperture in the mask was smaller than the active area of the dye-sensitized TiO_2_ (0.36 cm^2^). For complete masking, tape was also applied over the edges and rear of the cell. Current density–voltage (*J*–*V*) measurements were made by irradiating from behind with a LOT Quantum Design LS0811 instrument (100 mW cm^−2^ = 1 sun at AM 1.5) and the simulated light power was calibrated with a silicon reference cell.

### Electrolyte preparation

For DSCs with dye **1**, electrolyte compositions are given in Table 2 and Table 4. BMII and TBP were purchased from Sigma-Aldrich, and DMPII, BMIMPF, EMIMPF, MBI, MPN were bought from TCI, Apollo Scientific, Fluorochem, Alfa Aesar and Fluka, respectively. MeCN was HPLC grade. For DSCs with N719 dye, the electrolyte comprised electrolyte E1.

### EIS measurements

A ModuLab^®^ XM PhotoEchem photoelectrochemical measurement system from Solartron Analytical was used for the EIS measurements. The impedance was measured at the open-circuit potential of the cell at a light intensity of 22 mW cm^−2^ (590 nm) in the frequency range of 0.05 Hz to 100 kHz using an amplitude of 10 mV. The impedance data were analysed using the ZView^®^ software from Scribner Associates Inc.

## Supporting Information

Tables S1–S4: Additional tables of parameters for masked DSCs; Figure S1: EQE spectra; Figure S2: Equivalent electric circuit used to model EIS data; Figures S3–S5: DSC stability tests; Figure S6: Dependence of *V*_OC_ of DSCs on additive.

File 1 Further experimental data.
