# Supplementary material for: Electrolyte tuning in dye-sensitized solar cells with N-heterocyclic carbene (NHC) iron(II) sensitizers
Source: Beilstein J Nanotechnol. 2018 Dec 21;9:3069–78. doi: 10.3762/bjnano.9.285 (PMC6317411; doi:10.3762/bjnano.9.285)
Supplement: File 1 — Further experimental data. [file Beilstein_J_Nanotechnol-09-3069-s001.pdf]

BEILSTEIN JOURNAL OF NANOTECHNOLOGY

Supporting Information

for

Electrolyte tuning in dye-sensitized solar cells with *N*-heterocyclic carbene (NHC) iron(II) sensitizers

Mariia Karpacheva, Catherine E. Housecroft and Edwin C. Constable

Beilstein J. Nanotechnol. **2018,** *9,* 3069–3078. doi:10.3762/bjnano.9.285

Further experimental data

License and Terms: This is a supporting information file under the terms of the Creative Commons Attribution License (http://creativecommons.org/licenses/by/4.0). Please note that the reuse, redistribution and reproduction in particular requires that the authors and source are credited.
The license is subject to the *Beilstein Journal of Nanotechnology* terms and conditions: (https://www.beilstein-journals.org/bjnano)

Table S1: Parameters for duplicate masked DSCs using electrolytes with different ionic liquids with MPN and MeCN as solvent. Measurements were made on the day of sealing the cells. Data are referenced with respect to a DSC with N719, and the Rel. η values and with respect to η for N719 set to 100%.

Electrolyte	Cell	J_{SC} [mA cm^{-2}]	V_{OC} [mV]	ff [%]	η [%]	Rel. η [%]
E1	1	0.32	429	72	0.10	1.67
E1	2	0.34	451	73	0.11	1.8
E2	1	0.54	427	71	0.17	2.8
E2	2	0.54	426	71	0.16	2.6
E1a	1	0.19	419	68	0.05	0.8
E1a	2	0.24	418	68	0.07	1.2
E2a	1	0.25	432	69	0.07	1.2
E2a	2	-	-	-	-	-
E3	1	0.24	477	69	0.08	1.3
E3	2	0.29	480	73	0.10	1.6
E4	1	0.74	519	69	0.26	4.3
E4	2	0.70	529	70	0.26	4.3
E4	3	0.90	538	69	0.33	5.5
E4	4	0.82	542	69	0.31	5.1
N719	-	13.87	705	62	6.02	100

Table S2: Parameters for duplicate masked DSCs using different additives and additive concentrations (see Table 3) in the electrolytes. Measurements were made on the day of sealing the cells. Data are referenced with respect to a DSC with N719, and the Rel. η values and with respect to η for N719 set to 100%.

Electrolyte	Cell	J_{SC} [mA cm^{-2}]	V_{OC} [mV]	ff [%]	η [%]	Rel. η [%]
E1b	1	1.94	304	64	0.38	6.3
E1b	2	2.90	261	47	0.36	6.0
E3b	1	2.13	308	56	0.37	6.1
E3b	2	1.64	343	62	0.35	5.8
E4b	1	0.74	43	26	0.01	0.2
E4b	2	0.83	50	27	0.01	0.2
E2c	1	0.69	395	65	0.18	2.9
E2c	2	0.54	393	64	0.14	2.3
E2d	1	1.31	395	60	0.31	5.1
E2d	2	1.22	358	58	0.25	4.2
E2f	1	0.39	368	66	0.09	2.6
E2f	2	0.69	387	67	0.18	2.9
E2g	1	1.16	380	65	0.29	4.8
E2g	2	1.09	372	66	0.27	4.5
E2h	1	0.76	541	62	0.26	4.3
E2h	2	0.73	518	64	0.24	4.0
N719	1	13.87	705	62	6.02	100

Table S3: Parameters for multiple masked DSCs using different additives and additive concentrations (see Table 3) in the electrolytes. Measurements were made on the day of sealing the cells. Data are referenced with respect to a DSC with N719, and the Rel. η values and with respect to η for N719 set to 100%.

Electrolyte	Cell	J_{SC} [mA cm^{-2}]	V_{OC} [mV]	ff [%]	η [%]	Rel. η [%]
E2b	1	2.58	292	63	0.47	7.8
E2b	2	2.38	326	65	0.51	8.5
E2b	3	2.31	339	65	0.51	8.5
E2b	4	2.34	374	65	0.57	9.3
E2e	1	2.60	310	62	0.50	8.3
E2e	2	2.51	308	63	0.49	8.1
E2e	3	2.78	307	62	0.53	8.8
E2e	4	2.61	315	62	0.51	8.5
N719	1	13.87	705	62	6.02	100

Table S4: Parameters for masked DSCs using electrolytes E2b and E2e over 40 days.

Electrolyte	Cell	Day	J_{SC} [mA cm^{-2}]	V_{OC} [mV]	ff [%]	η [%]
E2b	1	0	2.58	292	63	0.47
		5	2.54	401	52	0.53
		14	2.19	425	58	0.54
		25	1.85	446	63	0.52
		40	1.35	469	67	0.43
E2b	2	0	2.38	326	65	0.51
		5	2.32	403	60	0.56
		14	2.17	421	59	0.54
		25	2.07	457	62	0.59
		40	1.73	460	64	0.51
E2b	3	0	2.34	374	65	0.57
		5	2.15	428	62	0.57
		14	2.04	441	61	0.55
		25	1.88	469	64	0.57
		40	1.53	470	66	0.48
E2e	1	0	2.51	308	63	0.49
		5	2.16	385	63	0.53
		14	1.95	418	65	0.53
		25	1.76	436	66	0.50
		40	1.46	446	66	0.43
E2e	2	0	2.78	307	62	0.53
		5	2.17	387	61	0.52
		14	1.73	435	64	0.48
		25	1.32	461	68	0.41
		40	0.90	466	71	0.29
E2e	3	0	2.61	315	62	0.51
		5	2.19	379	61	0.50
		14	1.97	418	63	0.52
		25	1.70	437	65	0.48
		40	1.32	446	67	0.40

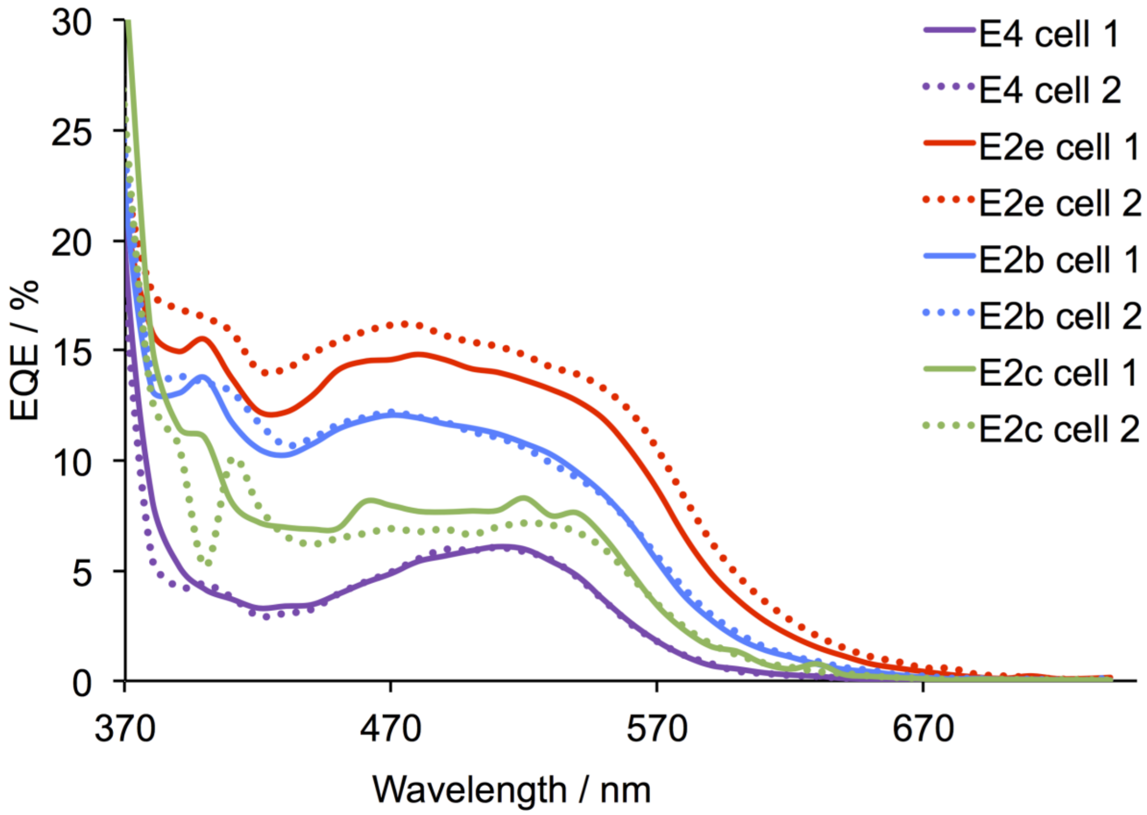

Figure S1: EQE spectra for duplicate DSCs with dye **1** and electrolytes E4 and E2b, E2c, E2e recorded on the day of sealing the DSCs.

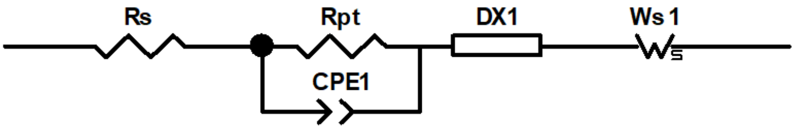

Figure S2: Equivalent electric circuit used to model EIS data.

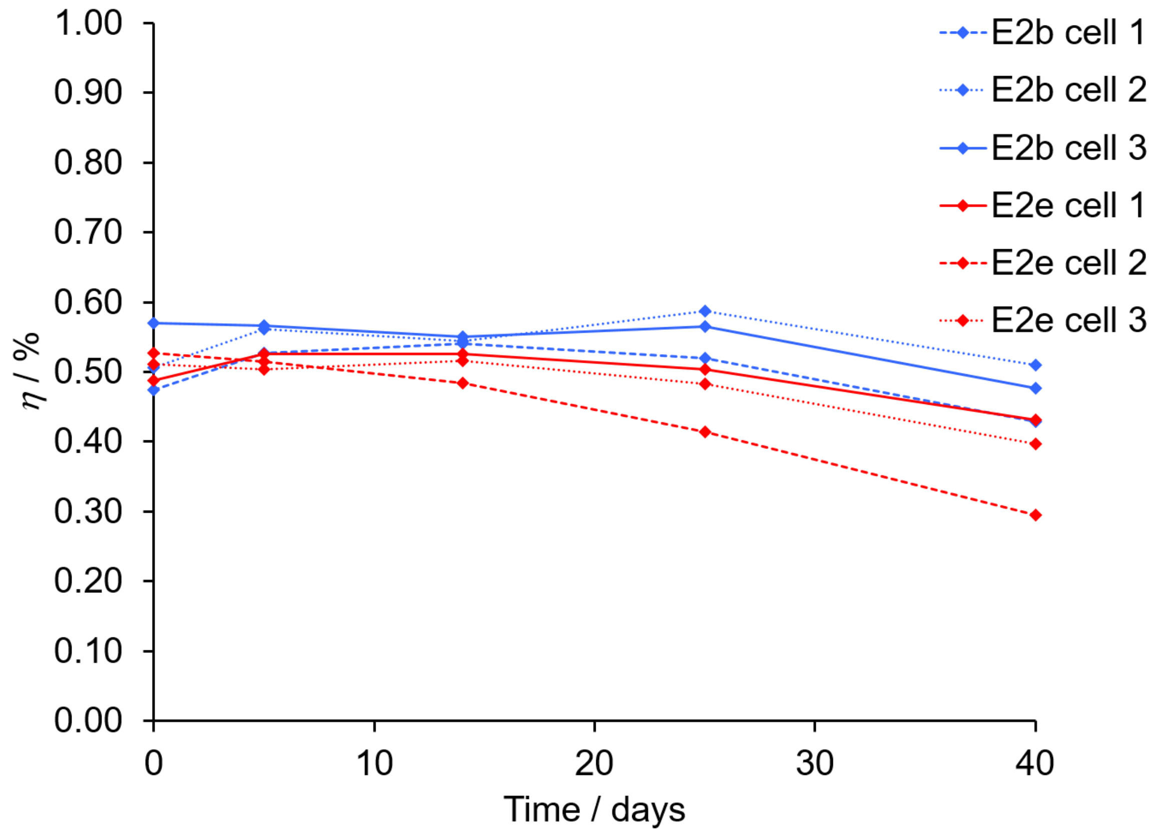

Figure S3: Variation in overall DSC efficiencies over a 40 day period for DSCs with electrolytes E2b and E2e.

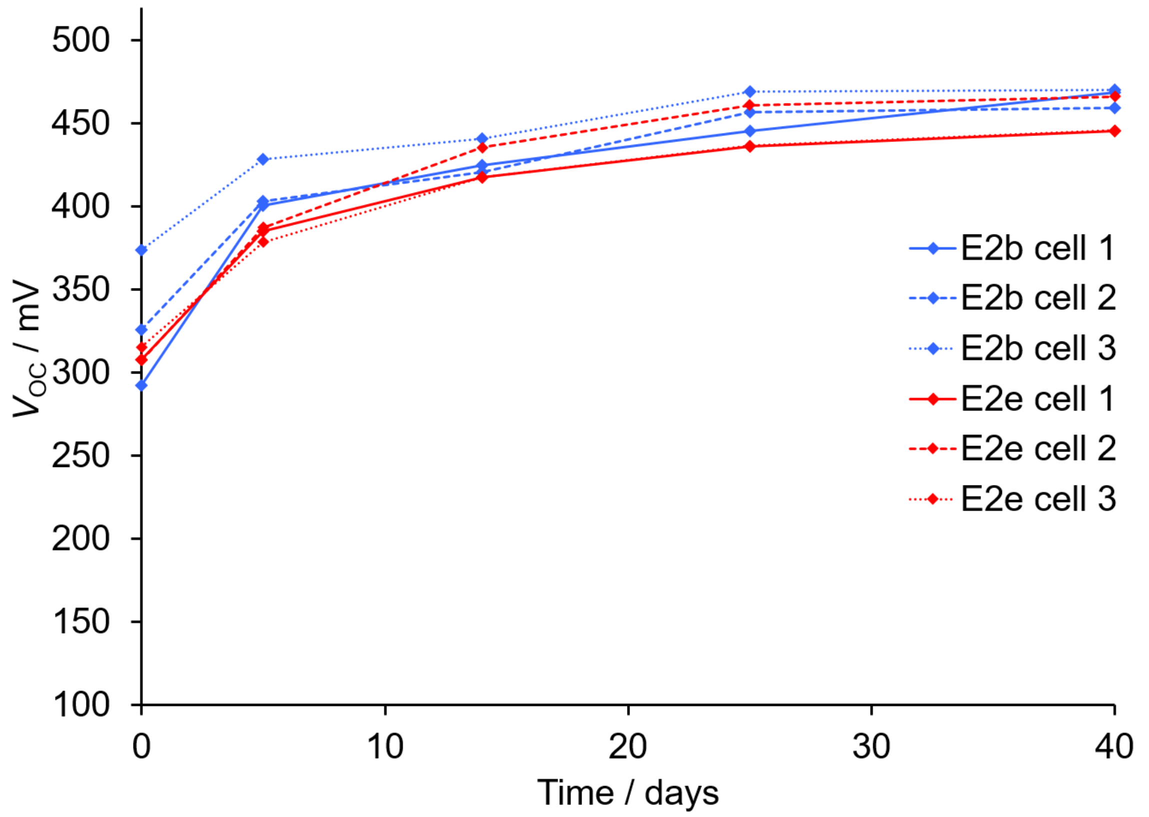

Figure S4: Variation in values of V_{OC} over a 40 day period for DSCs with electrolytes E2b and E2e.

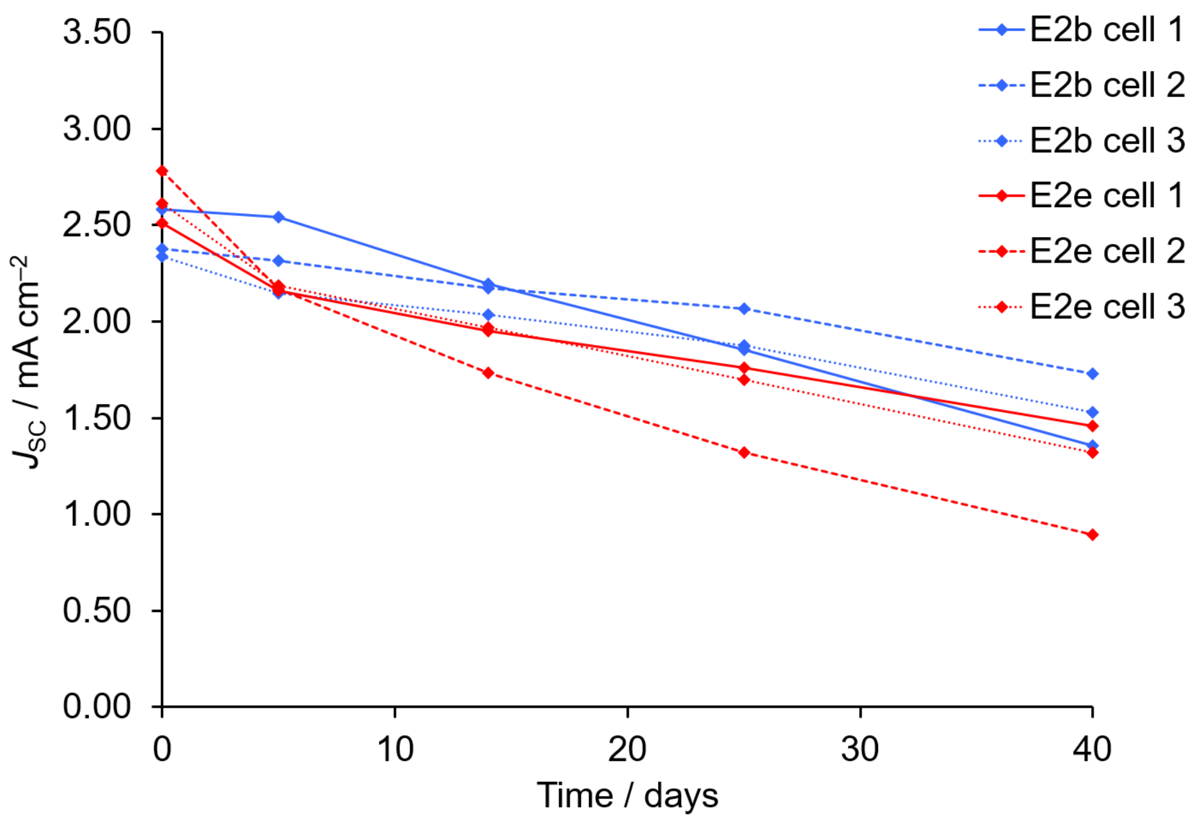

Figure S5: Variation in values of J_{SC} over a 40 day period for DSCs with electrolytes E2b and E2e.

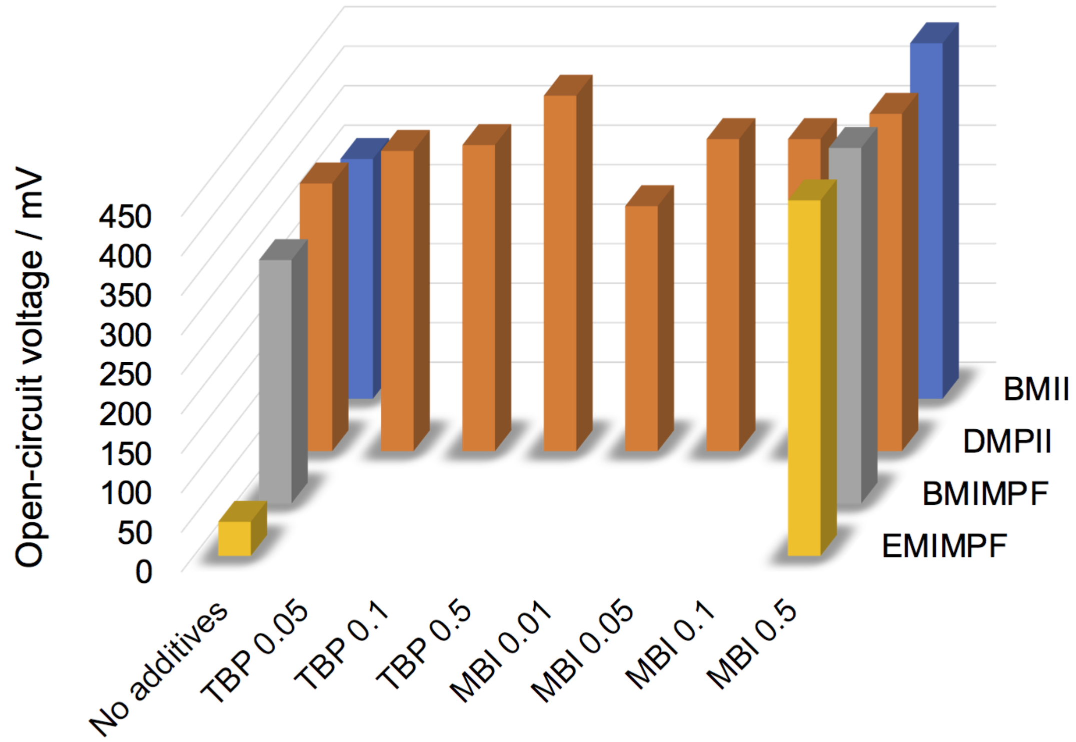

Figure S6: The dependence of open-circuit voltage of the DSCs (on day of sealing the cells) on additives (concentrations in M). Electrolyte compositions are LiI (0.1 M, I_2 (0.05 M, ionic liquid (0.6 M) in MPN with additives as specified on the abscissa.
